# Effect of antioxidants on the shear bond strength of composite resin to enamel following extra-coronal bleaching 

**DOI:** 10.4317/jced.55359

**Published:** 2019-02-01

**Authors:** Diatri Nari-Ratih, Andina Widyastuti

**Affiliations:** 1 MDSc., MClinDent (Endodontist), Ph.D. Department of Conservative Dentistry, Faculty of Dentistry, Universitas Gadjah Mada, Yogyakarta, Indonesia; 2 MClinDent (Endodontis). Department of Conservative Dentistry, Faculty of Dentistry, Universitas Gadjah Mada, Yogyakarta, Indonesia

## Abstract

**Background:**

Recently patients need faster treatments, and delaying restoration is not possible following bleaching treatment. The purpose of this study was to determine the effect of antioxidants, namely 10% sodium ascorbate, 10% alpha-tocopherol, 10% green tea and 10% Aloe vera extract on the shear bond strength of composite resin to enamel following extra-coronal bleaching using 40% hydrogen peroxide.

**Material and Methods:**

Seventy premolars were randomly assigned into 7 groups of 10 each. Group 1: bleaching treatment and no antioxidants application. Group 2: composite was built-up immediately after bleaching. Group 3: bleached specimens received composite build-up delayed by 2 weeks. Group 4, 5, 6 and 7: bleached specimens received an application of 10% sodium ascorbate, 10% alpha-tocopherol, 10% green tea, and 10% Aloe vera before composite build-up. Specimens were immersed in artificial saliva, stored in an incubator 37°C (24 hours), thermocycling, and tested using a universal testing machine. Data were analyzed by one-way ANOVA and Tukey’s test with 95% level of significance.

**Results:**

Bleaching caused significantly reduced shear bond strength (*p*<0.05), and application of 10% sodium ascorbate, 10% alpha-tocopherol, 10% green tea and 10% Aloe vera produced significantly greater shear bond strength compared to bleached group (*P* <0.05). However, no significant differences occurred between antioxidant groups (*P* >0.05).

**Conclusions:**

Application of antioxidants increased the shear bond strength of composite resin to enamel following extra-coronal bleaching using 40% hydrogen peroxide. 10% sodium ascorbate, 10% alpha-tocopherol, 10% green tea and 10% Aloe vera extracts produced the same effect on the shear bond strength of composite resin to enamel following extra-coronal bleaching using 40% hydrogen peroxide.

** Key words:**Antioxidants, shear bond strength, composite resin, extra-coronal bleaching.

## Introduction

Increasing interest in esthetic dentistry has resulted in the widespread practice of extra-coronal bleaching or vital bleaching. Extra-coronal bleaching is considered a safe, popular, conservative and well-accepted treatment modality for discolored teeth (1). Bleaching agents in varying concentration, namely carbamide peroxide (35% to 37%) or hydrogen peroxide (30% to 40%) have been used to achieved rapid esthetic results. Furthermore, hydrogen peroxide and carbamide peroxide have been used successfully for many years to achieve lighter and more desirable tooth color (2). Hydrogen peroxide undergoes ionic dissociation to give rise to the formation of free radicals such as nascent oxygen, hydroxyl radical, per-hydroxyl, and superoxide anions when they are applied to dental structure (3). These free radicals are highly reactive and hence reach out for electron-rich regions of pigment inside the dental structure, breaking down the large pigmented molecules with conjugated double bonds involving carbon, nitrogen, and oxygen atoms into smaller, less pigmented ones (4).

However, numerous studies have shown that bleaching can cause complications that may vary from postoperative sensitivity to pulpal irritation, tooth structure alterations or microleakage of existing restorations (5-7). Another important complication following bleaching procedure is decreased bond strength of composite resin to enamel when bonding is performed immediately after the bleaching process; this is attributed to the presence of residual peroxide that interferes with resin adhesion and inhibits resin polymerization (8).

Some techniques have been suggested to solve the clinical problems related to post bleaching compromised bond strength, such as treated bleached enamel with alcohol before restoration, removal of the superficial layer of enamel, and the use of adhesives containing organic solvents (7,9). However, the general approach is to postpone any bonding procedure for a period after bleaching, because the reduction in bond strength has been shown to be temporary (10). The waiting period for bonding procedures after bleaching has been reported to vary from 24 hours to 4 weeks (11). Therefore, to overcome this delay in restorative treatment, several studies have proposed the use of antioxidant agents namely 10% sodium ascorbate (7,8), and 10% alpha-tocopherol (12,13), after the bleaching procedure.

Sodium ascorbate is a neutral, nontoxic, and biocompatible antioxidant that when used as a 10% solution and application time of 10 min can reverse the reduced bond strength of bleached enamel (14). Alpha-tocopherol is the most active component of the vitamin E complex and is a powerful antioxidant in the human body in the lipid phase. The critical role of alpha-tocopherol protecting against free-radical reactions becomes apparent when considering the vast number of diseases and conditions, such as aging, many types of cancer, atherosclerosis and other circulatory diseases, arthritis, cataract formation, senile dementia, and respiratory diseases induced by pollution that are thought to be caused by these reactions (9).

Recently other natural products are used for antioxidant agents such as green tea and Aloe vera extracts, which have free radical scavenging ability that are more potent than sodium ascorbate and alpha-tocopherol (15). The green tea is made from the Camellia sinensis plant. It contains flavanols or catechins, such as epicatechin (EC), gallocatechin (GC), epigallocatechin (EGC), epicatechin gallate (ECG), and epigallocatechin gallate (EGCG). Green tea catechins have shown to possess potent antioxidant activity. In recent years, the use of green tea has been studied in dentistry. Previous investigators reported that use of green tea decreases dentin erosion and anti-microbial agents and increases enamel bonding strength values after bleaching (16). Additionally, green tea is a natural product, cheap and with an extended shelf life and could be an option for use as antioxidant agent following bleaching (14).

It has been postulated that the polysaccharides in Aloe vera gel have therapeutic properties such as immune-stimulation, anti-inflammatory effects, wound healing and anti-oxidant effects (17). Since Aloe vera grows abundance in Indonesia, then this material can be developed as an antioxidant after bleaching.

However, no studies have been conducted to compare the effects of sodium ascorbate and alpha-tocopherol to green tea and Aloe vera extracts on the bond strength of bleached enamel. Hence, the aim of this in vitro study was to evaluate and compare the effects of 10% sodium ascorbate, 10% alpha-tocopherol, 10% green tea and 10% Aloe vera extracts on the bond strength of enamel following extra-coronal bleaching using 40% hydrogen peroxide. The following null hypotheses were tested in this study: there would be no effect of 10% sodium ascorbate, 10% alpha-tocopherol, 10% green tea, and 10% Aloe vera extracts on the shear bond strength of composite resin restoration to enamel following extra-coronal bleaching.

## Material and Methods

This study was approved by Faculty of Dentistry Universitas Gadjah Mada Research Ethics Committee. Seventy extracted human premolars were collected, and stored in a distilled water following extraction. All teeth used in this study were extracted in the course of 1 months. The roots were sectioned approximately 2 mm apically of cemento-enamel junction (CEJ) using microtome (Diamond saw, Maruto, Japan). Each crown of teeth then was cut in mesial distal direction, and the buccal side of crowns were used in this study. Buccal side of crowns were embedded in an acrylic resin (Hillon S, Court Limited, England) block, keeping only the buccal portion exposed, and were flattened with 600 grit silicon carbide paper ((Moyco Precision Abrasives, Montgomeryville, PA, USA) to obtain flat and rough enamel surfaces. All specimens were observed under light microscope to verify enamel exposure rather than dentin occurred.

All specimens were assigned randomly into 7 groups of 10 each. Group 1 (served as control), no bleaching treatment and no antioxidant application, specimens were immersed in artificial saliva for 2 weeks. Group 2, specimens were bleached using 40% hydrogen peroxide (Opalescence Xtra Boost 40%, Ultradent, South Jourdan, UT, USA) as manufacturer’s direction. After bleaching, composite resin restorations were performed immediately. Specimens were acid etched with 37% phosphoric acid (DeTrey Conditioner 36, Dentsply deTrey, Konstanz, Germany) for 20 seconds, rinsed for 30 seconds and air dried for 10 seconds. A thin layer of adhesive material (XP Bond, Dentsply deTrey) was applied on the etched enamel, gently spread with compressed air and light-cured for 10 seconds. The embedded specimens were mounted in an apparatus containing a split metal mold with a circular hole 3 mm in diameter and 4 mm in height. Two increments of a composite resin (Ceram X Duo, Dentsply deTrey) were inserted into the hole of the split mold and each increment was light cured for 20 seconds. Therefore, composite resin restoration attached to the buccal portion of crown. The specimens were stored in an artificial saliva (Faculty of Natural Science, Universitas Gadjah Mada) for 24 hours in 37ºC incubator.

Group 3, specimens were bleached as group 2, then were immersed in the artificial saliva for 2 weeks. After immersion, specimens were restored using composite resin as group 2. Group 4, specimens were bleached as in group 2, specimens were applied 10% sodium ascorbate (Sodium L-ascorbate, Sigma Aldrich, Germany) as an antioxidant. Group 5, specimens were bleached as group 2, then were applied 10% alpha-tocopherol (Sigma Aldrich, Germany). To attain 10% sodium ascorbate, 10 g cristal of sodium ascorbate was dissolved in distilled water, and 10 g alpha-tocopherol was diluted in ethyl ethanol to make 10% solutions. Group 6, specimens were bleached as group 2, then were applied 10% green tea leaf extract (Faculty of Pharmacy, Universitas Gadjah Mada). Group 7, specimens were bleached as group 2, then were applied 10% Aloe vera extract (Faculty of Pharmacy, Universitas Gadjah Mada). Green tea leaf extracts were prepared by maceration method with ethanol solvent and were dilutes using distilled water to obtain 10% concentration (18). Same method was undertaken to make 10% Aloe vera extracts). After application of antioxidants, group 4, 5, 6 and 7 were restored using composite resin as group 2. To apply antioxidants on each specimen was used syringe and 0,02 mL of antioxidants were applied on to enamel surface using sponge pellet once/minute for 10 minutes. All enamels were then rinsed using distilled water for 30 seconds each.

**Figure 1:**
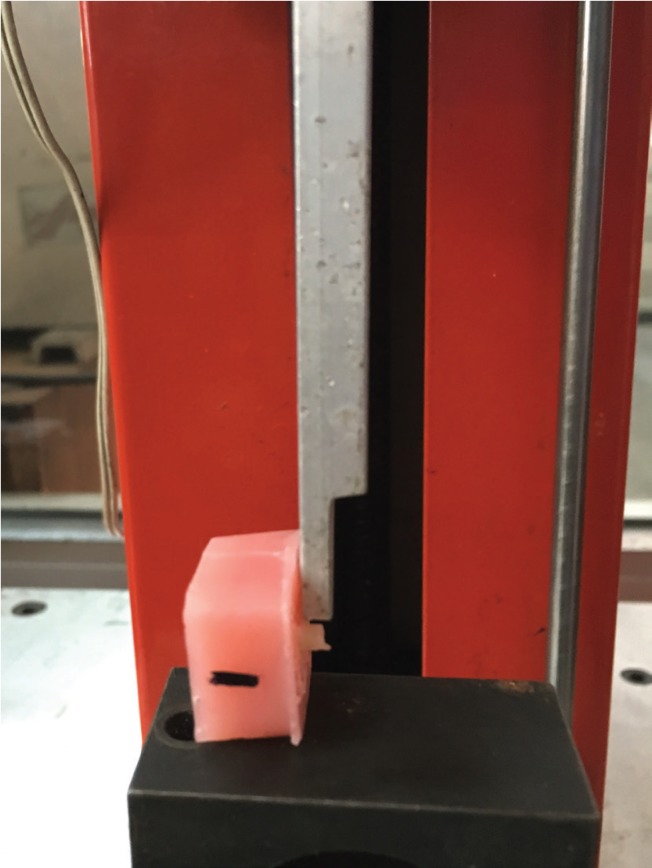
The knife edge was loaded at the interface between the composite and enamel surface.

Each specimen was loaded in universal testing machine for shear bond strength testing (Pearson Parke Equipment Ltd., London, UK). The long axis of the specimen was perpendicular to the direction of the applied forces. The knife edge was loaded at the interface between the composite and enamel surface (Fig. 1). The shear bond strength was measured in shear mode at a crosshead speed of 1 mm/min until fracture occurred. The results were expressed in MPa, and were analyzed using a one-way ANOVA, followed by Tukey’s test at the 5% level of significance. Fracture analysis of the bonded enamel surface was performed using a scanning electron microscopy (SEM) at a magnification of X1000 (Jeol JSM T300, Tokyo, Japan). The types of fractures were considered and classified as adhesive (lack of adhesion), cohesive (failure of the tooth substrate or composite resin) or mixed (adhesive and cohesive failures) (12).

## Results

One-way ANOVA showed significant differences in shear bond strength among the groups (*p*<0.05). Results revealed ( Table 1) that specimens were restored using composite resin immediately following bleaching, had the lowest shear bond strength compared to other groups (*p*<0.05). In contrast, group 1 (control), which was unbleached, produced the highest shear bond strength compare to other groups, but this value of shear bond strength was almost similar to group 3, which was bleached and had a waiting period for 2 weeks before composite resin restoration (*p*>0,05). Among antioxidants groups, 10% alpha-tocopherol revealed the highest shear bond strength compare to other antioxidants groups, however the differences were not significant (*P*>0.05), whereas 10% Aloe vera extract produced the lowest shear bond strength compared to other antioxidant groups (*P*>0.05).

**Table 1:**
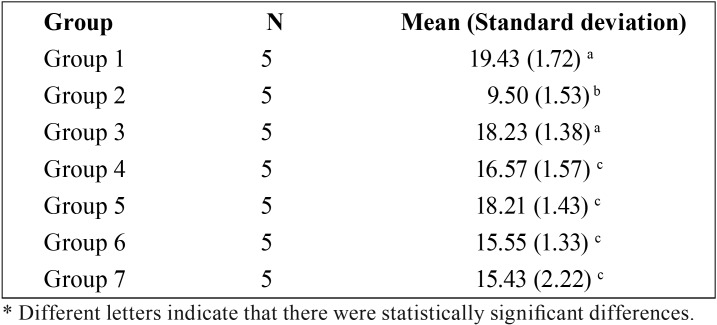
Group 1. No bleaching, Group 2. After bleaching, composite resin restorations were performed immediately. Group 3. Bleaching followed by immersion in the artificial saliva for 2 weeks. Group 4. Bleaching followed by 10% sodium ascorbate application. Group 5. Bleaching followed by 10% alpha-tocopherol application. Group 6. Bleaching followed by 10% green tea extract. Group 7. Bleaching followed by 10% Aloe vera extract.

**Figure 2:**
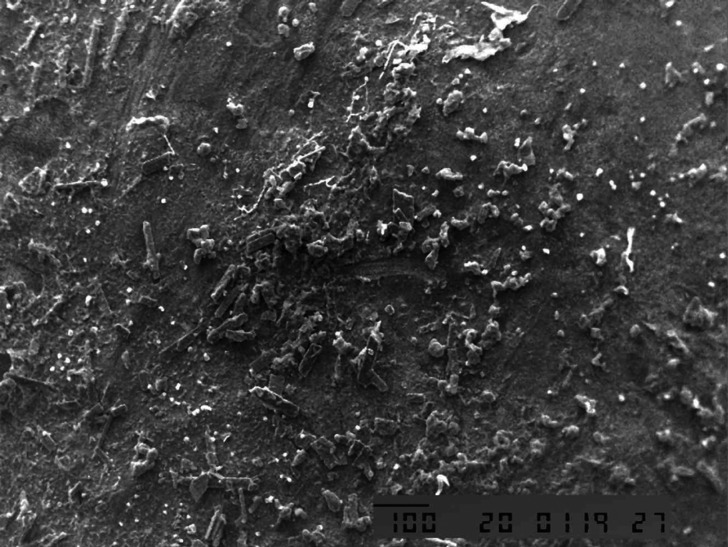
SEM photomicrographs showing adhesive type of failure.

**Figure 3:**
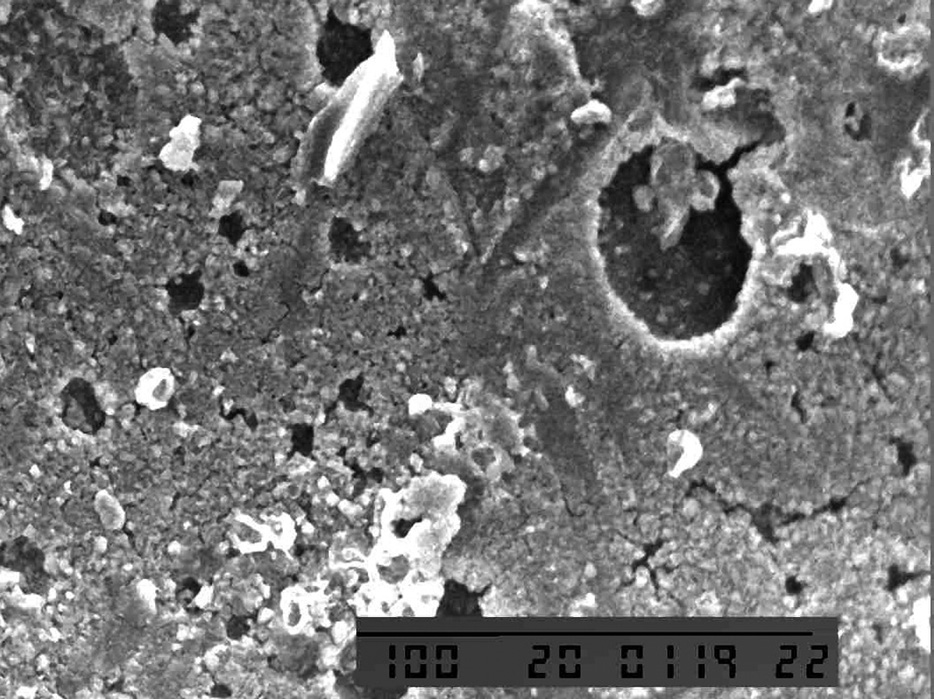
SEM photomicrographs exhibiting mixed failures between adhesive and cohesive types.

Scanning electron microscopy observation of the fractured specimens demonstrated that the majority of failure of the specimens, which were restored immediately using composite resin following bleaching, were adhesive type (Fig. 2). In contrast the majority of specimens, which were applied antioxidants before restoration, produced mixed failures between adhesive and cohesive types (Fig. 3).

## Discussion

Extra-coronal bleaching procedures are the most commonly used conservative and effective treatment options to treat discolored teeth (3). In the present study, it was observed that the extra-coronal bleaching procedure using 40% hydrogen peroxide resulted in a significant decrease of bond strength values compared to the unbleached group. The bleaching agents release free radicals as the nascent oxygen and hydroxyl or peri-hydroxyl ions when they are applied to the dental structure. Free radical is any molecule that has one unpaired electron, providing it high reactivity. These molecules are able to react with the electron-rich regions of the pigments inside the dental structure, breaking down large pigmented molecules into smaller, less pigmented ones (1,2). On the other hand, this property could be deleterious to the bonding of resinous materials. One theory proposed to explain the influence of bleaching agents on bonding suggests that peroxides and their by-products present inside the dental structure are capable to interfere with the polymerization process of the adhesive material (19).

Furthermore, Whang and Shin (9) evaluated the scanning electron microscope (SEM) images of interfaces between resin and bleached enamel and observed fragmented and poorly refined resin tags that penetrated to a lesser depth when compared with unbleached controls. In addition, the entrapment of peroxide ions into the bleached enamel resulted in a resin-bleached enamel interface that was granular and porous with a bubbled appearance. The alteration in organic substance, loss of calcium, and a decrease in micro-hardness added to this effect (11). Thus, antioxidant agents have been studied with the aim of inactivating free radicals.

The bleached specimens in the group 2, which were immediately restored using composite resin without any antioxidant treatment showed the lowest bond strength values with bond failure at the interface between the tooth substrate and the bonding agent. This was probably due to residual oxygen produced by bleaching agent on the tooth surface inhibiting polymerization of the bonding agent. As a result, the oxygen-rich tooth structure did not provide a good surface for bonding (20). In addition, as reported by Moosavi *et al.* (2), the effect of the bleaching agent reached the inside of the tooth structure. Whang and Shin (9) observed using SEM, revealed that 40% hydrogen peroxide produces a much more irregular pattern on dentin, with shallow erosive areas covering the sample surface. However, when the sodium ascorbate solution was applied to the tooth surface, the bond strength of the adhesive to the bleached tooth surface was maintained at a level equivalent to that of non-bleached surfaces.

It has been shown that bleaching treatment with hydrogen peroxide, hydroxyl radicals in the apatite lattice are substituted by peroxide ions, resulting in the formation of peroxide-apatite. After a two-week waiting period, peroxide ions may decompose and substituted hydroxyl radicals re-enter the apatite lattice, resulting in elimination of the structural changes caused by the incorporation of peroxide ions (20). Therefore, the results showed that waiting period of 2 weeks produced the almost similar value of shear bond strength compared to control. Furthermore, Sharafeddin *et al.* (8) reported that dentin and dentinal fluid can act as a peroxide and oxygen reservoir. The reservoirs of gaseous or dissolved oxygen products could persist until removed by pulpal micro-circulation and diffusion from the external surface. A greater surface diffusion would be expected based on a reduced pulpal microcirculation. Thus, levels of peroxide or oxygen higher than normal may be present in bonding interface, inhibiting the polymerization reaction and reducing bond strengths (5,11,21).

Antioxidant agents may facilitate free radical polymerization of the adhesive resin without the occurrence of early termination (22). Neutralizing process from antioxidant agents to free radicals is categorized into 3 types, i.e., the prevention of continuous (full-time prevention), active detoxification of oxidative stress, and passive detoxification. Sodium ascorbate, alpha-tocopherol, green tea and Aloe vera are included in passive detoxification that can neutralize free radicals and belong to non-enzyme antioxidants (23). However, none of the antioxidant agents was capable of completely neutralizing the deleterious effects of bleaching on bond strength.

Sodium ascorbate is a sodium salt of ascorbic acid and a well-known antioxidant. This agent is also capable of reducing a variety of oxidative compounds, particularly free radicals. Ascorbic acid also shows high antioxidant activity. However, its pH is approximately 1.8, which makes it inappropriate for clinical use. In contrast, sodium ascorbate has a pH of 7.4, but its antioxidant activity is similar to that of ascorbic acid (9). The antioxidizing ability of sodium ascorbate aided to neutralize and reverse the oxidizing effects of the bleaching agent (20). Therefore, the altered redox potential of the oxidized bonding substrate is restored and polymerization of the adhesive continues without permanent termination (14). The results of this present study support these findings. The antioxidant concentration of 10% was employed in this study due to the effective concentration used for antioxidant to neutralize the free oxygen present in a higher amount in dentin than in enamel as reported by previous study (12).

Vitamin E is the term used for a group of tocopherols and tocotrienols, of which alpha-tocopherol has the highest biological activity. Vitamin E functions as a chain-breaking antioxidant that prevents propagation of free radical reactions (24). It has been used on dentin and enamel with good bonding results and it also shows the antioxidant activity similar to that of ascorbic acid (9). Some studies reported that alcohol application on bleached enamel increased bond strength, although the values did not return to the levels of the nonbleached group (12). The presence of alcohol in the composition of the 10% α-tocopherol solution formulated for this study may have contributed to the good response in reversing the compromised bond strength of bleached enamel since 10% α-tocopherol was not miscible in water solutions (25). Thus, the phenomenon observed might be not only due to the antioxidant agent of α-tocopherol but also to the presence of alcohol (26). Furthermore, vitamin E is more oxidizing and stable than ascorbate because of its hydrophobicity.

The results also demonstrated that green tea extract reversed the reduction in shear bond strength although it has lower shear bond strength value compared to sodium ascorbate and alpha-tocopherol. It can be explained that the green tea catechins, such as EGCG and EGC, have potent antioxidant activities caused by the three adjacent OH groups on the B-ring that scavenge free radicals more effectively than the two adjacent OH groups in ECG and EC (8,11). Thus, green tea catechins were shown to possess potent antioxidant activity. In this way, it was speculated that EGCG can be the main responsible for the capture of free radicals from the bleaching (15). Further chemical and analytical studies must be conducted to elucidate the mechanism by which green tea reverses the bond strength of enamel after bleaching. It was speculated that higher concentrations of green tea may result in a greater reversal of bond strength values in bleached enamel.

The antioxidant effect of Aloe vera due mainly to polysaccharides found in parenchyma tissue of Aloe vera leaves, and other substances containing in Aloe vera leaves namely polyphenols, indoles, and alkaloids. This material can neutralize the effect of oxygen residue on the enamel surface by bleaching, therefore Aloe vera extract could reverse the shear bond strength of composite resin restoration following bleaching (27). According to Barandozi (28), antioxidant activity of Aloe vera is not only caused by a single chemical component but the action of several components containing in Aloe vera that work synergistically.

The failure surface observed using SEM was in accordance with the results of this study that revealed that majority of specimens, which were applied antioxidants showed mixed failure between adhesive and cohesive. Conversely, specimens, which were restored composite immediately revealed adhesive failure. This phenomenon indicates that application of antioxidants increases the bond strength of the composite restoration (29).

The results of this present study showed that sodium ascorbate and alpha-tocopherol produced non-significant greater shear bond strength than green tea and Aloe vera extracts (*p*>0.05). This condition is probably caused by lower molecular weight in sodium ascorbate and alpha-tocopherol, although in this study molecular weight was not measured of each antioxidant agent. Consequently, the lower molecular weight of sodium ascorbate and alpha-tocopherol cause these antioxidants to penetrate deeply into enamel than green tea and Aloe vera extracts (4).

## Conclusions

Within the limitation of this study, it can be concluded that application of antioxidants increased the shear bond strength of composite resin to enamel following extra-coronal bleaching using 40% hydrogen peroxide. 10% sodium ascorbate, 10% alpha-tocopherol, 10% green tea and 10% Aloe vera extracts produced the same effect on the shear bond strength of composite resin to enamel following extra-coronal bleaching using 40% hydrogen peroxide.
